# Polygenic risk for obsessive-compulsive disorder (OCD) predicts brain response during working memory task in OCD, unaffected relatives, and healthy controls

**DOI:** 10.1038/s41598-021-98333-w

**Published:** 2021-09-23

**Authors:** Stephan Heinzel, Christian Kaufmann, Rosa Grützmann, Julia Klawohn, Anja Riesel, Katharina Bey, Stefanie Heilmann-Heimbach, Leonie Weinhold, Alfredo Ramirez, Michael Wagner, Norbert Kathmann

**Affiliations:** 1 Department of Psychology, Humboldt-Universität zu Berlin, Rudower Chaussee 18, 12489 Berlin, Germany; 2 Clinical Psychology and Psychotherapy, Department of Education and Psychology, Freie Universität Berlin, Habelschwerdter Allee 45, 14195 Berlin, Germany; 3 Department of Psychology, University of Hamburg, Von-Melle-Park 11, 20146 Hamburg, Germany; 4 Department of Psychiatry and Psychotherapy, University Hospital Bonn, Venusberg-Campus 1, 53127 Bonn, Germany; 5 German Center for Neurodegenerative Diseases (DZNE), Venusberg-Campus 1, 53127 Bonn, Germany; 6 Institute of Human Genetics, School of Medicine and University Hospital Bonn, Venusberg-Campus 1, 53127 Bonn, Germany; 7 Institute for Medical Biometry, Informatics and Epidemiology, University Hospital Bonn, Venusberg-Campus 1, 53127 Bonn, Germany; 8 Division of Neurogenetics and Molecular Psychiatry, Department of Psychiatry and Psychotherapy, Faculty of Medicine and University Hospital Cologne, University of Cologne, Kerpener-Str. 62, 50931 Cologne, Germany; 9 Department of Psychiatry & Glenn Biggs Institute for Alzheimer’s and Neurodegenerative Diseases, San Antonio, TX USA; 10 Cluster of Excellence Cellular Stress Responses in Aging-associated Diseases (CECAD), University of Cologne, Cologne, Germany; 11 Department of Neurodegenerative diseases and Geriatric Psychiatry, University Hospital Bonn, Medical Faculty, Venusberg-Campus 1, 53127 Bonn, Germany

**Keywords:** Genetics, Neuroscience, Psychology, Biomarkers, Risk factors

## Abstract

Alterations in frontal and parietal neural activations during working memory task performance have been suggested as a candidate endophenotype of obsessive-compulsive disorder (OCD) in studies involving first-degree relatives. However, the direct link between genetic risk for OCD and neuro-functional alterations during working memory performance has not been investigated to date. Thus, the aim of the current functional magnetic resonance imaging (fMRI) study was to test the direct association between polygenic risk for OCD and neural activity during the performance of a numeric n-back task with four working memory load conditions in 128 participants, including patients with OCD, unaffected first-degree relatives of OCD patients, and healthy controls. Behavioral results show a significant performance deficit at high working memory load in both patients with OCD and first-degree relatives (p < 0.05). A whole-brain analysis of the fMRI data indicated decreased neural activity in bilateral inferior parietal lobule and dorsolateral prefrontal cortex in both patients and relatives. Most importantly, OCD polygenic risk scores predicted neural activity in orbitofrontal cortex. Results indicate that genetic risk for OCD can partly explain alterations in brain response during working memory performance, supporting the notion of a neuro-functional endophenotype for OCD.

## Introduction

Obsessive-compulsive disorder (OCD) is a relatively frequent mental disorder (2–3% of the population^1^) that is characterized by intrusive and unwanted obsessive thoughts and compulsive behaviors^2^. As indicated by family studies, first-degree relatives (REL) have a four to five-fold risk to develop OCD compared to the general population^3,4^. Genetic factors are important in the etiology of OCD and obsessive-compulsive symptoms as indicated by heritability estimates. A recent meta-analysis of genome-wide association studies (GWAS) reported an estimated common single-nucleotide polymorphism-(SNP-)based heritability of 28% for OCD, which is among the highest found for neuropsychiatric disorders. While no SNPs reached genome-wide significance, polygenic risk scores based on the OCGAS or IOCDF-GC GWAS significantly predicted case–control status in the other study, respectively^5^. In current studies, a significant genetic correlation between OCD and sub-syndromal obsessive-compulsive symptoms was found^6,7^, and polygenic risk scores based on the IOCDF-GC GWAS predicted obsessive-compulsive symptoms in a large population-based sample^8^.

In order to understand the etiologic pathways from genotype to OCD phenotype, the investigation of intermediate phenotypes (endophenotypes) has become a useful research strategy^9^. Endophenotypes are characterized by their heritability and deviant expressions in both OCD patients and unaffected relatives of patients.

Dysfunctions in executive control, (i.e. cognitive flexibility and working memory updating) have been proposed as a possible endophenotype for OCD (for review see^10^). Performance deficits in executive control have been reported both in patients with OCD and REL^11,12^. Functional neuroimaging studies during executive control tasks have shown OCD-related functional alterations mainly in orbitofronto-striatal and fronto-parietal networks^4,13^. In the context of working memory, these dysfunctions have been related to deficient updating and short-term maintenance of information in OCD^14–16^. However, the majority of research has been performed only in patients with OCD in comparison to healthy control subjects, thus, it appears premature to determine whether the neural alterations presented in these studies actually represent an endophenotype for OCD or a consequence of the disorder and/or the treatment. To address this issue, studies that also included unaffected REL^17–19^ have been conducted. In fact, unaffected REL showed deviant brain activity in fronto-parietal regions (most pronounced in dorsolateral prefrontal cortex (DLPFC) and pre-supplementary motor area) compared to healthy controls (HC) during a working memory updating task^17^. While these findings suggest that genetic variations may cause the working memory-related alterations in frontal and parietal regions, a more direct evidence for this notion is still missing. To date, there is no study investigating relationships between neuroimaging data obtained during a working memory task and genetic (i.e. DNA sequence) data in OCD and unaffected REL. Therefore, the current investigation for the first time seeks to assess the impact of genetic variation on working memory-related brain function in groups of patients with OCD, unaffected REL, and HC.

Previous morphometric imaging genetics studies have identified several core regions that were associated with candidate genes related to the serotonergic, dopaminergic, and glutamatergic systems (for review see^20,21^). Gene variants of the serotonergic and glutamatergic system affected grey matter volume in orbitofrontal cortex (OFC). Glutamatergic gene variations also affect anterior cingulate cortex and thalamus. Dopaminergic genes were found to influence activity in putamen. Due to the complexity and heterogeneity of the OCD phenotype, current large consortia such as the ENIGMA (Enhancing Neuro Imaging Genetics through Meta-Analysis) consortium are focusing on combining polygenic risk with neuroimaging^22^.

In the present study, we aimed to test the hypothesis of deviant neural activity in brain regions of the orbitofronto-striatal and fronto-parietal circuits during working memory updating being a potential endophenotype for OCD. This hypothesis would be supported, if (a) orbitofronto-striatal and/or fronto-parietal alterations in blood oxygen level-dependent (BOLD) response were found in both OCD and unaffected REL, and (b) OCD polygenic risk scores would predict these neuro-functional deviations.

## Materials and methods

### Participants

Fifty-four patients with OCD, 37 unaffected first-degree relatives of OCD patients and 56 healthy control participants were recruited for the current study. Four healthy controls, two relatives, and two OCD patients had to be excluded from data analyses due to technical failures during fMRI scanning. Furthermore, three healthy controls, three relatives and one OCD patient showed performance at chance level (performance below 30% hit rate or above 30% false alarm rate) in the working memory task, and thus, had to be excluded from data analyses as well. Another four healthy controls received a post-hoc diagnosis in the SCID interview (two skin picking disorder, one anxiety disorder, one substance abuse disorder) and were excluded from the healthy control sample. Therefore, the final analysis sample consisted of 51 patients with OCD, 32 unaffected REL of patients with OCD, and 45 healthy control participants without a family history of OCD. Out of the 32 REL, 24 were parents of a patient with OCD, 5 were siblings, and 3 were children. Please note that only 7 out of 32 REL (22%) were related to a patient with OCD who participated in our study, and only 5 out of the 51 study participants with OCD (10%) were related to participants of our REL sample. Past and present mental disorders were assessed in all participants by trained clinical psychologists using the German version of the Structured Clinical Interview for DSM-IV TR (SCID^23^). Information on psychopathology of the relatives of all participants were obtained by the Family History Screen^24^. All participants were between 18 and 65 years of age, had normal or corrected-to-normal vision, reported no history of any neurological diseases or brain injuries, and were suitable for MRI scanning. OCD patients were recruited from the OCD outpatient clinic at Humboldt-Universität zu Berlin, Germany and were diagnosed with OCD as verified with the SCID. Patients were excluded if they had a current or lifetime diagnosis of psychotic, bipolar, or substance use disorder, or if they took neuroleptic medication in the past 4 weeks or benzodiazepines in the past 2 weeks. REL of patients with OCD were excluded if the SCID did not verify the diagnosis of their affected relative or if they had a current or lifetime diagnosis of OCD, psychotic, bipolar, or substance use disorder. REL were also excluded if they took any psychotropic medication in the past 4 weeks. Healthy control participants were recruited via online and public advertisements and were matched for age, gender, and education level to the OCD patients. The following exclusion criteria applied to HC: psychotropic medication in the past 3 months, any current or past mental disorder according to DSM-IV TR axis-I, family history of OCD. The study was approved by the local Ethics Committee of the Humboldt-Universität zu Berlin and conducted in accordance with the Declaration of Helsinki. Note that the current study is part of a larger project, and other results of this project have been published elsewhere^14,25–27^. All participants gave written informed consent after receiving written and verbal information about the study, and received a monetary compensation for their time.

### Genotyping and polygenic risk scores

DNA samples from blood (n = 112) or saliva (n = 7) were genotyped using the Infinium Global Screening Array (Illumina) at the LIFE and BRAIN GmbH Bonn, Germany. Genotype quality control was done using Plink-1.9, and R (version 3.5.1). We checked the data for sex inconsistencies and grossly failing markers (call rate < 0.5). Individuals with a call rate of < 0.95 were removed. The heterozygosity rate for each subject was calculated; outliers (± 3 SD from the mean heterozygosity rate) were identified and removed. On marker level, SNPs were removed if at least one of the following conditions was true: significant difference of missing rate between cases and controls, call rate < 0.95; deviation of Hardy–Weinberg equilibrium (*p* < 1 × 10^–6^); and minor allele frequency < 0.05 (computed separately in cases and controls). Furthermore, all A/T or C/G SNPs were removed. A genetic relatedness check was done and none of the OCD patients were related to any of the HC participants. To check and correct for population stratification (i.e. allele frequency differences between cases and controls due to systematic ancestry differences), principal component analysis was performed including all SNPs^28^. This method uses genome-wide genotype data to estimate principal component axes that can be used as covariates in subsequent association analyses to control for spurious associations due to ancestry differences. Thus, the first two principal components were included as covariates in all analyses that involved polygenic risk scores. The genotyped data were imputed on the Michigan Imputation Service using the 1000 Genomes Phase 3 (Version 5) reference panel. Low quality (INFO-score < 0.5) and rare (MAF < 0.01) variants were removed from the imputed data set, leaving 4,869,847 variants. The polygenic risk scores for each participant of our study were computed using PLINK^29^. For the polygenic score calculation, we used summary statistics from the Psychiatric Genomics Consortium (PGC) genome-wide association studies (GWAS) for OCD^5^ as a discovery sample. The number of risk alleles carried for each selected SNP (i.e., 0, 1, or 2) was weighted by the log(OR) provided by the PGC GWAS, and averaged across all SNPs. 5381 SNPs were selected that were significant at a significance threshold of p < 0.01. This significance threshold was chosen as these top-ranked SNPs were suggested to have an important role in gene expression in the brain and possibly in the etiology of OCD^30^. Absolute values of polygenic risk scores were z-transformed for further analyses. Note, that in our fMRI sample, OCD polygenic risk scores could be obtained from 119 subjects (43 HC, 32 REL, 44 OCD).

### N-back paradigm during fMRI

We used the same task setup as reported previously^14^: Sixteen blocks (4 blocks of each 0-, 1-, 2-, and 3-back) were presented in different pseudo-randomized orders. The working memory load condition of each block was indicated by a cue displayed 2 s before the block started. In each block, 16 randomly generated digits from 0 to 9 were presented in the center of a black screen one at a time for 500 ms with an interstimulus interval of 900 ms; the occurrence of 5 target stimuli was pseudo-randomized. Targets were defined as re-occurrence of a number previously presented 1, 2, or 3 trials before (1-, 2-, or 3-back condition). In the 0-back condition, the target was defined as the digit ‘0’. The participants were instructed to press a button with their right thumb when they recognized a target. After each block, a white fixation cross was presented in the center of a black screen for 4 s. Every fourth block, the fixation cross was presented for 14 s. The total task duration was 9:00 min. Before the fMRI session, two practice sessions of the n-back task were performed outside the scanner to familiarize participants with the task. The n-back task was presented using Presentation software (version 18.2, Neurobehavioral Systems Inc., Albany, CA, USA). N-back performance was defined as hit rate minus false alarm rate.

### MR image acquisition

FMRI data were collected at the Berlin Center for Advanced Neuroimaging, Charité Campus Mitte, Berlin, Germany with a 3 Tesla Magnetom Trio Tim MR system (Siemens, Erlangen, Germany). In the beginning of each scanning procedure, one T1-weighted 3D pulse sequence was obtained (repetition time (TR) = 2440 ms, echo time (TE) = 4.81 ms, flip angle = 8°, matrix size = 256 × 256, 192 sagittal slices with 0.91 mm thickness, voxel size = 0.91 × 0.91 × 0.91 mm^3^). Additionally, a T2-weighted 3D pulse sequence was applied (TR = 5000 ms, TE = 499 ms, flip angle = 120°, acquisition matrix = 256 × 258, 192 sagittal slices, with an isotropic voxel size of 0.91 mm). Functional data were obtained using a gradient echo-planar imaging (GE-EPI) pulse sequence (TR = 2000 ms, TE = 30 ms, flip angle = 78°, matrix size = 64 × 64, voxel size = 3.0 × 3.0 × 3.75 mm). 32 slices were acquired descending parallel to the bicommissural plane. See also^14^ for details on image acquisition.

### MR image processing and analysis

All fMRI analyses were carried out with SPM12 (revision 6906; Wellcome Trust Centre For Neuroimaging, London, UK). After correction for head motion and computation of a mean EPI image, the T1w image was co-registered to the mean EPI image and normalized (by integrating information of the T2w image) into the spatial standard space as defined by the template of the International Consortium for Brain Mapping (http://www.loni.ucla.edu/ICBM/). None of the participants had to be excluded due to excessive head movements. Spatial transformations as estimated during the segmentation procedure were applied to EPI images. EPI images were resampled into isotropic voxels with an edge length of 2 mm and spatially smoothed with an isotropic Gaussian kernel of 8 mm full width at half maximum^14^.

### Estimation of BOLD effects in n-back

The working memory experiment was analyzed within the framework of the General Linear Model (GLM). As described in previous work^14,31^, at the single subject level, we created design matrices comprising the experimental conditions of 0-, 1-, 2-, and 3-back as separate regressors of interest and all other experimental conditions (cue, button presses, and the six rigid body realignment parameters) as regressors of no interest. The GLM was fitted voxel-wise into the filtered time series using the restricted maximum likelihood algorithm as implemented in SPM12. Three contrasts of interest were built: 1-back > 0-back, 2-back > 0-back, and 3-back > 0-back. On the second level, a random effects model as implemented in the GLM_Flex_Fast4 toolbox http://mrtools.mgh.harvard.edu/index.php?title=GLM_Flex) was applied for a repeated measures ANCOVA with the between-subjects factor group (OCD vs. REL vs. HC), the within-subjects factor working memory load (1 > 0-back vs. 2 > 0-back vs. 3 > 0-back), and the covariate age. Age was included as a covariate to control for age differences between groups (see Table 1) and because of its effect on working memory performance and brain response^32,33^. Whole brain analyses of the group by working memory interaction effects were thresholded at p < 0.05, family-wise error (FWE) at cluster-level. Analyses were performed for the whole brain, restricted to gray matter according to the tissue probability map thresholded at 0.3 as implemented in SPM12. We used a Monte Carlo simulation correction (10,000 iterations) with an initial voxel-wise threshold of p < 0.001 (http://afni.nimh.nih.gov/pub/dist/doc/program_help/3dClustSim.html). Clusters with a minimum cluster size of 48 voxels yielded a cluster-level FWE threshold of p < 0.05 and are described in the results section and in Table 2.

**Table 1:** Demographics of HC (HC), patients with obsessive-compulsive disorder (OCD), and unaffected first-degree relatives of OCD patients (REL).

Measure	HC (N = 45)	REL (N = 32)	OCD (N = 51)	p (HC vs. REL)	p (HC vs. OCD)	p (OCD vs. REL)
Age	31.00 (7.56)	46.17 (14.77)	33.00 (9.73)	**< 0.001**	0.268	**< 0.001**
Sex	19 m/26 f	12 m/20 f	26 m/25 f	0.814	0.419	0.264
Verbal test score	32.13 (3.85)	32.50 (3.26)	31.20 (4.78)	0.663	0.297	0.178
Y-BOCS severity scale (sum)^a^			23.25 (5.21)			
Y-BOCS subdimension taboo			3.10 (2.77)			
Y-BOCS subdimension contamination			4.33 (3.37)			
Y-BOCS subdimension rituals			2.41 (2.48)			
Y-BOCS subdimension hoarding			4.47 (2.85)			
Y-BOCS subdimension doubt			4.12 (2.85)			
N with at least one comorbid Axis I disorder^b^			43			
Current medication^c^			22			
Polygenic risk score^d^	− 0.23 (1.02)	− 0.02 (1.10)	0.25 (0.85)	0.393	**0.019**	0.232
Performance 0-back	99.59 (1.27)	97.74 (8.27)	97.89 (5.07)	0.144	**0.031**	0.921
Performance 1-back	96.60 (5.97)	92.23 (14.79)	96.62 (5.41)	0.078	0.982	0.057
Performance 2-back	83.07 (14.90)	70.47 (17.64)	81.18 (15.22)	**0.001**	0.540	**0.004**
Performance 3-back	82.89 (18.88)	59.56 (21.62)	72.33 (23.82)	**< 0.001**	**0.019**	**0.016**

**Figure 1:**
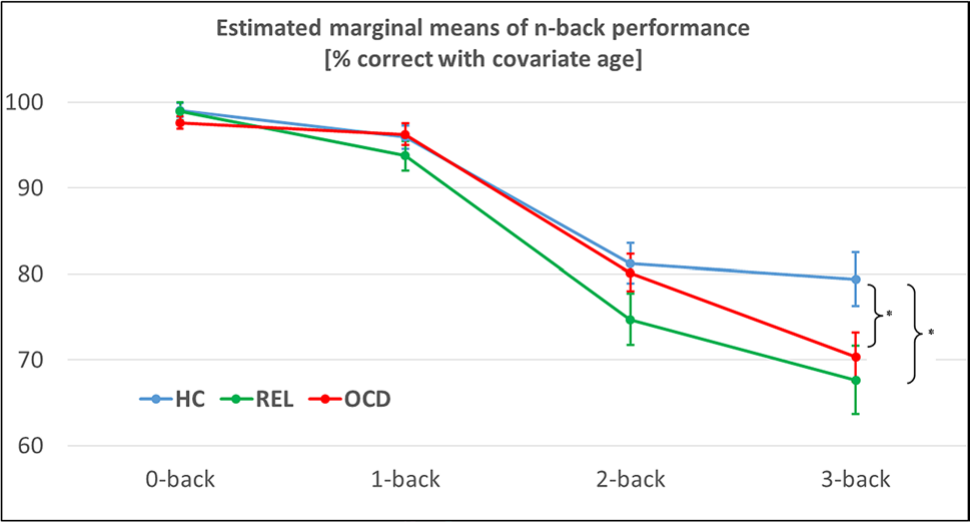
Behavioral n-back data in healthy controls (HC), patients with obsessive-compulsive disorder (OCD), and unaffected first-degree relatives of OCD patients (REL). Estimated marginal means are reported for performance [% correct] and are derived from the group (OCD vs. REL vs. HC) by working memory load (0- vs. 1- vs. 2- vs. 3-back) ANCOVA model with the covariate age. Error bars reflect standard errors of the mean. *p < 0.05.

Means and standard deviations (in parentheses) are shown. Reported p-values are derived from two-sample *t* tests or a χ^2^-test (for the variable sex). P-values with bold emphasis indicate significant differences at p < 0.05. Units: Age [years]; Verbal test score [sum score]; Y-BOCS [sum score]; Polygenic risk score [z-transformed values]; Performance [% correct], (see estimated marginal means from ANCOVA model with covariate age in Fig. 1).

^a^Subdimensions of Y-BOCS according to Katerberg et al.^57^.

^b^Comorbid mental disorders in OCD patients: 27 OCD patients had one comorbid mental disorder, 16 had two or more, and 8 had no comorbid mental disorder. In total, 44 mood disorders (23 currently remitted), 15 anxiety disorders, 3 eating disorders, 2 somatoform disorder, 1 tic disorder, 1 cannabis abuse were diagnosed.

^c^19 SSRIs, 4 SSNRIs, 5 tricyclic antidepressants, 2 neuroleptics, 1 benzodiazepine.

^d^In our fMRI sample, polygenic risk score was available in 43 HC, 32 REL, and 44 OCD.

**Table 2:** Group × working memory load interaction.

Region	Hem	MNI coordinates			t-value	cluster size
		x	y	z		
**(k > 48, p < 0.05 FWE cluster-corr.)**						
Superior parietal lobule/inferior parietal lobule/postcentral gyrus	L	− 24	− 48	68	9.30	1200
		− 56	− 32	42	8.56	Included
		− 28	− 40	46	7.76	Included
Inferior parietal lobule/intraparietal sulcus/supramarginal gyrus	R	54	− 32	50	9.15	413
		34	− 36	38	5.65	Included
		56	− 36	30	5.57	Included
Dorsolateral prefrontal cortex	L	− 48	2	32	7.40	82
Premotor cortex	L	− 42	0	52	6.57	55
Inferior frontal gyrus	L	− 48	8	14	6.21	52
Dorsolateral prefrontal cortex	R	38	2	36	6.18	55

Anatomical locations and MNI coordinates for the group (HC vs. patients with obsessive-compulsive disorder vs. unaffected first-degree relatives) by working memory load (1 > 0-back vs. 2 > 0-back vs. 3 > 0-back) interaction with the covariate age; whole-brain results are reported at p < 0.05, family-wise error (FWE) cluster-corrected (*Hem* hemisphere, *L* left, *R* right).

To assess the direct effect of OCD polygenic risk scores on BOLD response during n-back, a random effects model was applied to run a repeated measures ANCOVA with the within-subjects factor working memory load (1 > 0-back vs. 2 > 0-back vs. 3 > 0-back), and the covariates OCD polygenic risk score, the first two population structure principle components, and age. For this analysis, clusters with a minimum cluster size of 56 voxels yielded a cluster-level FWE threshold of p < 0.05.

### Statistical analyses of working memory performance, polygenic risk, and group status

Group differences in working memory performance were analyzed using a group (OCD vs. REL vs. HC) by working memory load (0- vs. 1- vs. 2- vs. 3-back) analysis of covariance (ANCOVA) model with the covariate age. To test associations between OCD polygenic risk scores and group status, an ordinal logistic regression was conducted. Linear regression analyses were performed to test associations between OCD polygenic risk scores and working memory performance. Note that, in line with previous reports including polygenic risk scores in related participants^34,35^, population structure covariates were included^28^ in all analyses that involved polygenic risk scores.

### Ethical standards

The authors assert that all procedures contributing to this work comply with the ethical standards of the relevant national and institutional committees on human experimentation and with the Helsinki Declaration of 1975, as revised in 2008.

## Results

### Behavioral results n-back

The three (group) by four (working memory load) ANCOVA with the covariate age of the n-back performance revealed a significant interaction of group by working memory load (F(6, 372) = 2.46, p = 0.024, partial η^2^ = 0.038) and a significant main effect of age (F(1, 124) = 19.54, p < 0.001, partial η^2^ = 0.136), as well as a significant interaction of working memory load by age (F(3, 372) = 10.17, p < 0.001, partial η^2^ = 0.076). Performance values are shown in Table 1, estimated marginal means of the ANCOVA model with the covariate age are shown in Fig. 1. Post-hoc two-sample *t* tests of this ANCOVA model indicated significant differences only in the 3-back condition between patients with OCD and HC (t(94) = 2.14, p = 0.035); and between REL and HC (t(75) = 2.35, p = 0.021). These results show that, when age is taken into account, HC show a higher performance in 3-back compared to both patients with OCD and REL. All other *t* tests were not significant (all p-values > 0.08).

### FMRI results during n-back

**Figure 2:**
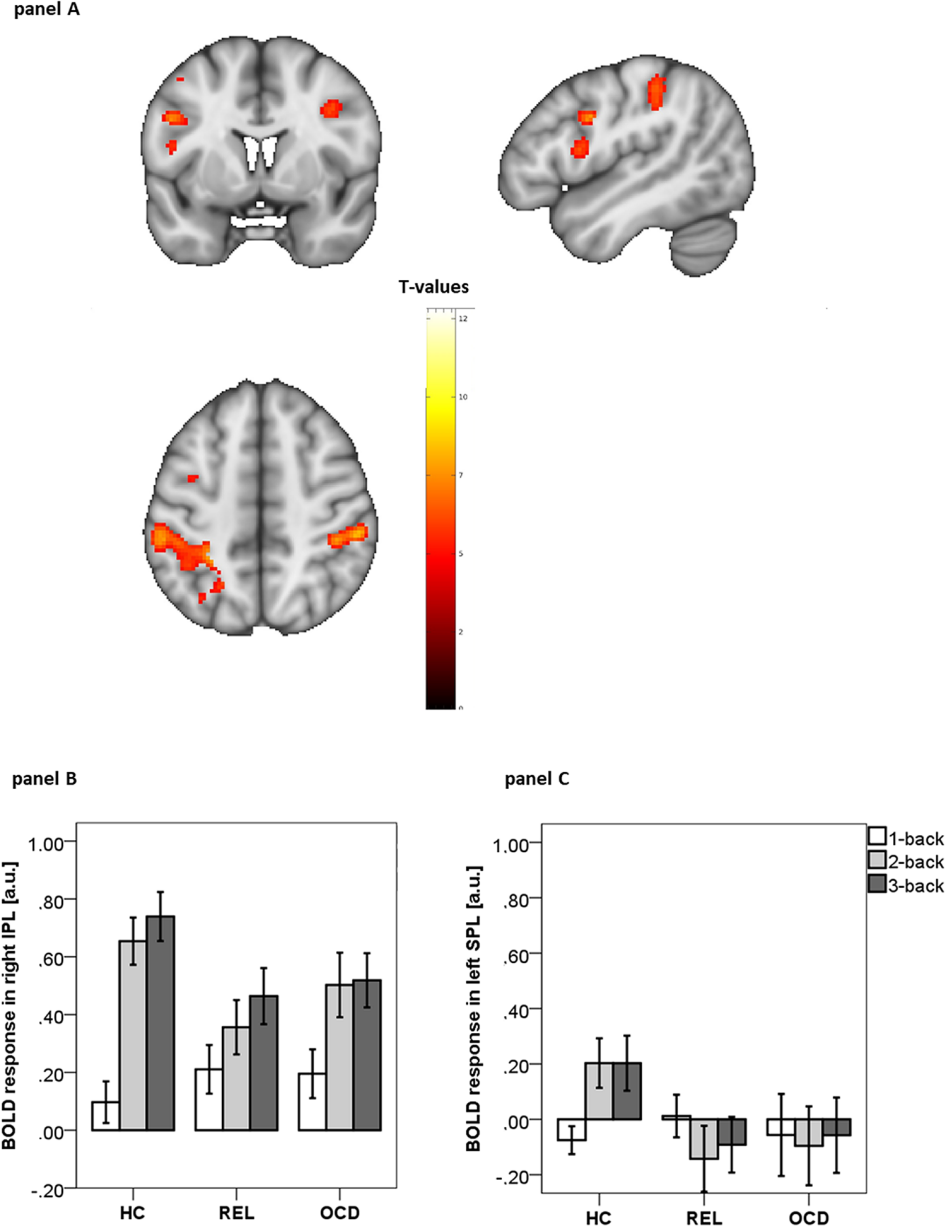
(**A**) Significant clusters of group (OCD vs. REL vs. HC) by working memory load (1 > 0-back vs. 2 > 0-back vs. 3 > 0-back) interaction, (p < 0.05 FWE cluster-corrected), color code: t-values; (**B**) Parameter estimates of BOLD response in arbitrary units [a.u.] for each group and working memory load in right IPL; (**C**) Parameter estimates of BOLD response in left SPL. Parameter estimates were obtained from a sphere (6 mm radius) around the peak voxels. MNI coordinates of peak voxels were for right IPL: -56 -32 42; for left SPL: -24 -48 68.

As shown in Fig. 2A, whole-brain analyses of the three (group) by three (working memory load) interaction revealed significant interaction effects in two large and four smaller clusters of the fronto-parietal working memory network (p < 0.05, FWE cluster-corrected). The highest t-values and largest cluster extents were found in left superior/inferior parietal lobule (left SPL/IPL, t = 9.30) and right inferior parietal lobule (right IPL, t = 9.15). The activation patterns (see Fig. 2B,C) indicate that both the OCD and the REL groups showed reduced activations for 2- and 3-back compared to the healthy control group. See Table 2 for significant clusters in bilateral dorsolateral prefrontal cortex (DLPFC), left inferior frontal gyrus (IFG), and left premotor cortex (PMC).

### OCD polygenic risk scores

OCD polygenic risk scores obtained from PGC-OCD data^5^ were significantly associated with group status (R^2^ = 0.043, F(1, 117) = 5.24, p = 0.024). As reported in Table 1, patients with OCD had the highest scores, REL had intermediate scores, and HC had the lowest scores.

No direct associations were found between OCD polygenic risk scores and n-back performance (p > 0.36) in the entire sample.

### OCD polygenic risk scores and BOLD response

**Figure 3:**
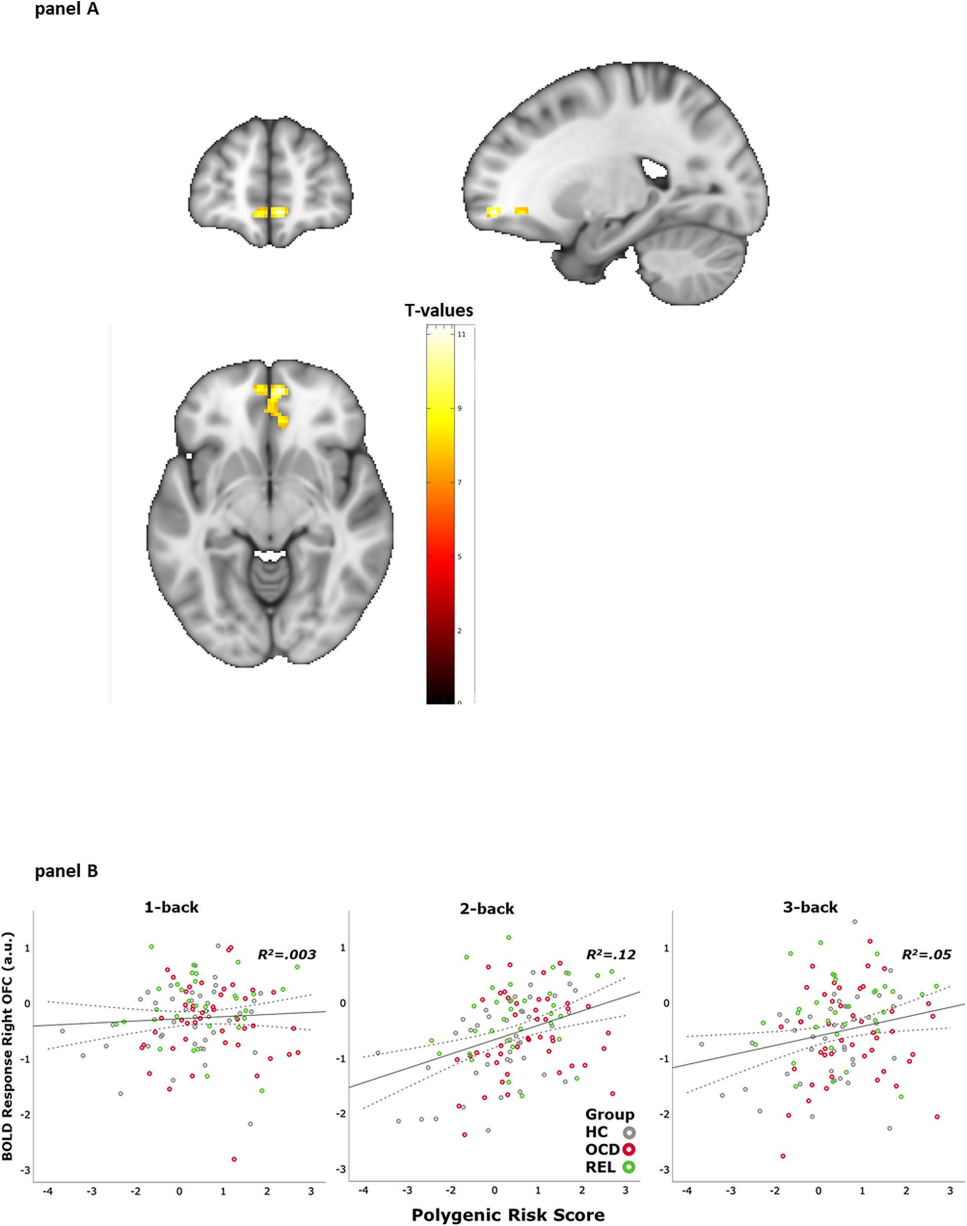
(**A**) BOLD response related to OCD polygenic risk scores from whole-brain ANCOVA with the within-subject factor working memory load (1 > 0-back vs. 2 > 0-back vs. 3 > 0-back) and the covariates OCD polygenic risk scores, the first two population structure principle components, and age (p < 0.05 FWE cluster-corrected); (**B**) R^2^ values represent the percentage of variance in BOLD response (in arbitrary units [a.u.]) for each working memory load in right medial OFC that was explained by the OCD polygenic risk scores [mean-centered values]. Parameter estimates were obtained from a sphere (6 mm radius) around the peak voxel (MNI coordinates: 6 54 − 8). Group status of the data points is color coded (HC: grey; OCD: red; REL: green).

As shown in Fig. 3A, the whole-brain ANCOVA with the within-subject factor working memory load and the covariates OCD polygenic risk scores, the first two population structure principle components, and age, revealed a significant effect for OCD polygenic risk scores in the right medial orbitofrontal gyrus (MNI coordinates 6 54 -8; t = 11.47, cluster size: 98 voxels, p < 0.05 FWE cluster-corrected). As shown in Fig. 3B, this finding indicates that higher OCD polygenic risk scores predicted an increase in BOLD response during 2- and 3-back but not during 1-back.

## Discussion

Results reflect that patients with OCD and unaffected REL showed performance decrements in working memory updating as well as reduced BOLD responses in right IPL, left SPL/IPL, bilateral DLPFC, left PMC, and left IFG during 2- and 3-back. Most importantly, OCD polygenic risk scores predicted BOLD response in medial OFC, indicating that higher genetic risk for OCD led to increased activity in medial OFC during the performance of a demanding working memory task.

Behavioral results are in line with previous reports of OCD-related impairments in executive functions (for review see^36,37^) and more specifically in working memory updating (for review see^38^). Our and previous studies that included both patients with OCD and unaffected REL, showed that executive dysfunctions can be seen not only in patients suffering from OCD, but also in subjects with a genetic risk for OCD^39,40^, thus supporting the notion of executive dysfunction being a candidate endophenotype of OCD.

The fMRI results from the group by working memory load analysis indicated that both patients with OCD and unaffected REL show reduced neural activity in the fronto-parietal working memory network^41^ during high task demand. Together with a marked performance decrement, these findings suggest an impaired functioning of the working memory system at high working memory load. It seems that these dysfunctions become visible when updating is required and may be due to inefficient strategies^38^. The strongest effects and largest cluster extents were found in the bilateral IPL/SPL. IPL was found to play an important role in working memory updating specifically involving selective attention, working memory rehearsal, and capacity^42,43^. As shown in the recently published mega-analyses from the ENIGMA-OCD working group^44^, reduced cortical thickness in bilateral IPL was the main OCD-associated structural brain imaging finding in their sample of 1498 adults with OCD. Thus, we had expected that IPL would also show altered activations during working memory performance as shown previously^17,45,46^.

In line with previous studies^17,45,47^, we also found altered activations in bilateral DLPFC, a region that has been associated with executive components of working memory (e.g. distractor resistance, updating, action selection^48^). However, in contrast to several previous studies that reported fronto-parietal hyperactivations during working memory performance^17,45,47^, we found mainly decreased activations at 2- and 3-back. These hypoactivations in bilateral IPL and DLPFC together with marked performance decrements in 3-back in OCD and REL, may indicate that compensatory attempts fail at high working memory load as suggested by models of an inverse U-shaped relationship between working memory load and BOLD responses^31,49,50^. These models indicate that impairments in the working memory system can be related to a left-ward shift of this function, showing relative hyperactivations at lower working memory load and hypoactivations at higher working memory load, as reported in OCD before^14,15^. This concept has been described in terms of a reduced fronto-parietal adaptability to increasing working memory load^14^ and may partially integrate different findings of previously reported hyper- and hypoactivations.

Crucially, polygenic risk for OCD predicted BOLD response during n-back performance in medial OFC, reflecting that neural activity in OFC increased with increasing genetic risk for OCD. Therefore, carrying a higher genetic risk to develop OCD seems to affect the medial OFC functioning during the execution of a demanding working memory task. It is important to note that this analysis is agnostic to phenotypical information such as OCD symptoms or OCD diagnosis. Thus, these results suggest that alterations in medial OFC functioning may play a role in the etiology of OCD as opposed to being a consequence of the OCD phenotype. Our study expands the literature on orbitofronto-striatal dysfunctioning that has previously described in terms of increased activity and connectivity of the OFC in the OCD phenotype^51–53^ by providing evidence for an association with the genetic risk for OCD. While previous genetic studies have reported effects of gene variants on OFC morphometry^20,21^, the current study showed that genetic risk for OCD may affect OFC functioning during a cognitively demanding task (n-back).

While reduced activity in lateral frontal areas and SPL/IPL was shown in the group by working memory load analysis for both OCD and REL, a correlation between SPL/IPL activity and OCD polygenic risk scores was only found in a small cluster that did not survive FWE-correction in the second model including OCD polygenic risk scores as predictors of BOLD response. Thus, the genetic contribution to alterations in lateral frontal cortex and IPL/SPL is less clear.

While OCD polygenic risk scores were significantly associated with group status, it explained only a relatively small portion of variance (R^2^ = 0.043). Since models including the factor group status also rely on information on OCD phenotype, diverging results between the two reported fMRI analyses are not surprising. They may reflect the importance of both genetic and environmental factors in the etiology of OCD, as well as limitations of the current mainly symptom-based classification systems for mental disorders^54^.

Since medial OFC is not considered to be a core region that is recruited during working memory performance in healthy subjects^41,48^, an increased neural activity in this region deviates from normal working memory-related activation patterns and has been associated with an OCD-related over-monitoring^16^. Over-monitoring that is applied during high working memory demand may become an inefficient strategy, eventually leading to impaired working memory performance.

Together with our findings of reduced lateral fronto-parietal functioning in OCD and REL of OCD-patients, results seem to point to a suggested OCD-related imbalance in cortico-basal ganglia-thalamo-cortical (CBGTC) circuits^52^. Thus, the over recruitment of a “limbic” CBGTC circuit involving medial OFC^55,56^ may interfere with the fronto-parietal functioning required for high working memory performance^17^.

The novel finding of the current study is a direct association between polygenic risk for OCD and neural activity in medial OFC, providing a new puzzle piece for the understanding of the etiology of OCD.

Some limitations of the study need to be noted. Groups differed in age, however, results were corrected for age differences and both behavioral and fMRI results remained significant when controlling for age. Since we aimed to investigate a naturalistic patient sample, the OCD group was relatively heterogeneous regarding medication, symptom dimensions, and comorbidity. Also, relatively large age differences add another level of heterogeneity to the sample. Our findings suggest that alterations in neural activity during working memory performance may apply to OCD patient populations in everyday care and are not restricted to highly selected study populations. Even though our sample size is the largest sample of OCD-patients and REL performing a working memory task during fMRI measurements to date, the sample is small for polygenic risk score analyses. Thus, our results need to be interpreted as preliminary and require replication in larger samples that would also facilitate further analyses such as comparisons between specific symptom dimensions or comorbidities.

Taken together, the results of the current study suggest that both patients with OCD and unaffected REL show a reduced activity in the fronto-parietal working memory network at high working memory load accompanied by deficient performance. The magnitude of genetic risk for OCD predicted the intensity of neural activity in the medial OFC agnostic to information on OCD symptoms or OCD phenotype, thus supporting the concept of a neuro-functional endophenotype for OCD.
